# Machine Learning-Driven Flexural Performance Prediction and Experimental Investigation of Glass Fiber-Reinforced Polymer Bar-Reinforced Concrete Beams

**DOI:** 10.3390/polym17060713

**Published:** 2025-03-07

**Authors:** Muhammet Karabulut

**Affiliations:** 1 Department of Structural Mechanics and Analysis, Technical University of Berlin, 10623 Berlin, Germany; m.karabulut@campus.tu-berlin.de or karabulut@beun.edu.tr; 2 Department of Civil Engineering, Zonguldak Bulent Ecevit University, 67100 Zonguldak, Turkey

**Keywords:** machine learning (ML), three-point bending test, glass fiber reinforced polymer (GFRP) bar, reinforced concrete beam, brittle, ductile, shear crack, flexural crack, ada boost regressor, gradient boosting regressor

## Abstract

This study experimentally examines the flexural performance, crack formation patterns, and failure mechanisms of glass fiber-reinforced polymer (GFRP) bar-reinforced concrete beams with varying concrete compressive strengths (low, moderate, and high), addressing a gap in the current literature. Furthermore, it employs an innovative machine learning approach to enhance analysis. Nine RC beams reinforced with GFRP bars, having concrete compressive strengths of low (CC20), moderate (CC30), and high (CC40), each measuring 150 × 200 × 1100 mm, were fabricated and tested under three-point bending conditions. Through the integration of three-point bending tests and machine learning-based prediction models, this study connects experimental findings with advanced analytical approaches. One of the key innovations in this study is the use of eighteen ML regression models implemented with Python’s PyCaret library, achieving an impressive average prediction accuracy of 91.5% for RC beam deflection values. In particular, the Ada Boost Regressor and Gradient Boosting Regressor models performed exceptionally well on GFRP bar-reinforced concrete beams, providing the highest number of consistent and highly accurate predictions, making them very useful tools for GFRP bar-reinforced beam ultimate load-carrying capacity/deflection predictions. The outcomes identified clear failure mechanisms: RC beams with CC20, CC30, and CC40 concrete compressive strengths typically developed a single, large flexural crack at the midpoint. Although the ultimate load-carrying capacity of GFRP bar RC beams improved with higher concrete compressive strength, CC20 and CC30 beams displayed more ductile failure behavior than CC40 beams. The ultimate load-carrying capacity of CC40 RC beams was determined to be approximately 74% higher than that of CC20 RC beams. Regardless of the concrete compressive strength class, the absence of shear cracks and the prevention of sudden failure under bending in GFRP bar-reinforced concrete beams are considered major advantages of using GFRP bar reinforcement.

## 1. Introduction

Recently, there has been a growing focus on finding alternatives to steel reinforcement in reinforced concrete structures, which dominate the construction industry. The primary motivations behind this search are the drawbacks of steel, including its heaviness, vulnerability to moisture and salt water, and the loss of strength over time due to corrosion. At this stage, composite bars stand out as an innovative and future-oriented alternative due to their corrosion resistance against moisture and salt water, lightweight nature, and superior tensile strength. GFRP composite bars were selected due to their cost-effective performance. The bending performance of GFRP bar-reinforced concrete beams was evaluated through three-point bending tests on nine beams with varying concrete compressive strength: low (C20), moderate (C30), and high (C40). With the advancement of cutting-edge technology and artificial intelligence (AI), it is believed that integrating these innovations into the construction sector will bring significant benefits, improving efficiency and accelerating project timelines. Another key aspect that makes this study significant is the prediction of the bending performance of GFRP bar-reinforced concrete beams based on experimental data using machine learning (ML) methods. The deflection values of the beams under ultimate load-carrying capacity, based on various data, were analyzed using 18 different machine learning regression models. The study achieved an average success rate of 87.2% in predicting the RC beams deflection behavior.

Research on the bond performance between glass fiber-reinforced polymer (GFRP) bars and ultra-high performance concrete (UHPC) was carried out [1,2]. According to research results, the bond strength of GFRP bars decreases as the diameter increases. This supports the idea that future studies should focus on using numerous smaller-diameter GFRP bars for reinforcement rather than relying on large-diameter GFRP bars.

An experimental and theoretical study was conducted on the flexural behavior of GFRP and CFRP bar-reinforced concrete beams after exposure to high temperatures [3]. The mechanical properties of FRP-reinforced high-strength concrete structures after exposure to high temperatures can be preliminarily predicted, providing a new theoretical foundation for the application of FRP-reinforced concrete in structural design.

The determination of the residual stiffness of polymeric-reinforced concrete beams under repeated loads was investigated [4]. This study utilizes 12 flexural elements with varying reinforcement and loading configurations to demonstrate the efficiency of the proposed methodology in quantifying the residual strength of the tension concrete, which in turn estimates the effectiveness of the reinforcement system.

An investigation was carried out on the shear behavior of concrete beams strengthened with FRP reinforcements and stirrups, utilizing a hybridized Artificial Neural Network (ANN) combined with a Genetic Algorithm [5]. The analytical results indicated that the predictions closely aligned with the test results, demonstrating reasonable accuracy. The model can, therefore, be effectively used to predict the shear capacity of concrete beams reinforced with FRP bars and stirrups.

A reliability-based design analysis for FRP-reinforced compression yield beams was investigated [6]. The study further highlights that, compared to FRP concrete beam designs, the ductile failure mode of compression-yielding beams permits a reduced safety factor to satisfy safety standards.

Simplified rules for serviceability control of fiber-reinforced polymer-reinforced concrete elements (FRPRC) were explored [7]. The results of parametric calculations for the slenderness limits of FRPRC elements are presented in the form of a diagram for various concrete classes.

The structural performance of lightweight aggregate concrete reinforced with glass or basalt fiber-reinforced polymer bars was investigated [8]. The findings of this study can further support the adoption of innovative materials like lightweight aggregates (LWA) and FRP reinforcement in construction.

A new analytical model for the deflection of concrete beams reinforced with BFRP bars and steel fibers under cyclic loading was studied [9]. The results indicate that steel fibers significantly enhanced both the ultimate moment capacity and the service load moment of the BFRP-reinforced concrete beams.

The flexural performance of concrete beams reinforced with continuous FRP bars and discrete steel fibers under cyclic loads was studied [10]. The outcomes indicate that an increase in the reinforcement ratios effectively controlled crack widths, deflections, and residual deformations, while also improving the flexural bearing capacity of the beams.

Ductility estimation for flexural concrete beams longitudinally reinforced with hybrid FRP–steel bars was investigated [11]. The tests revealed that the flexural capacity of beams with hybrid reinforcement was 15–45% higher than that of beams with conventional steel bars, depending on bar positions and the ratio of longitudinal reinforcement.

The flexural behavior of natural hybrid FRP-strengthened reinforced concrete (RC) beams and strain measurements using Brillouin Optical Time Domain Analysis (BOTDA) were studied [12]. The analytical outcomes for the control beam specimen closely matched the experimental results, especially regarding the maximum deflection.

The environmental impacts of glass and carbon fiber-reinforced polymer bar-reinforced seawater and sea sand concrete beams used in marine environments were studied [13]. When taking into account the increased transportation distance and enhanced durability performance, the advantages of GFRP/seawater and sea sand concrete (SWSSC) and CFRP-SWSSC beams become more pronounced.

The preloading effect on the strengthening efficiency of reinforced concrete (RC) beams strengthened with non- and pre-tensioned near-surface mounted (NSM) FRP strips was investigated [14]. Experiments revealed promising outcomes under both ultimate and serviceability limit state conditions. A significant increase in load-bearing capacity, ranging from 56% to 135% compared to the unstrengthened beams, was achieved.

Glass fiber-reinforced polymer (GFRP) reinforced concrete beams were investigated in terms of shear strength using the finite element method and experimentally [15,16,17,18]. Studies on GFRP bar-reinforced concrete beams have highlighted that the finite element method produced results consistent with experimental findings, underscoring the effectiveness of finite element analysis in future research.

The shear behavior of FRP bar-reinforced deep beams was explored [19,20]. Crack paths, failure loads, and stress values are presented and discussed. Flexural and deflection behaviors of GFRP-reinforced concrete beams were examined [21,22,23,24]. Recycled aggregate, engineered cementitious composite, regular oriented fibers and GFRP bars, and synthetic FRP under sustained service load topics were investigated.

The tensile behavior of concrete reinforced with macro fibers processed from waste GFRP composites was examined [25]. The relationship between tensile stress and crack opening was investigated.

Although the use of machine learning methods in structural engineering has been studied very limitedly in the literature compared to traditional methods, it has yielded satisfactory and highly consistent results. The nonlinear load-deflection analysis of concrete beams reinforced with steel reinforcement was investigated using a machine learning method [26]. Deflection values of reinforced concrete beams could be predicted with an impressive average accuracy of 95%. It is suggested that ML models should also be investigated for FRP-reinforced concrete beams. A study was conducted to explore the use of machine learning in predicting the flexural strength of concrete beams reinforced with a combination of steel and FRP bars [27]. The outcomes display that the XGBoost algorithm delivers high accuracy in predicting the bending capacity of RC beams.

Researchers investigated the use of advanced hybrid machine learning models to predict the compressive and tensile strength of basalt fiber-reinforced concrete [28]. The proposed hybrid models indicated strong effectiveness in predicting compressive strength and tensile strength, highlighting their potential for estimating the durability characteristics of basalt fiber-reinforced concrete. The exploration focused on data-driven models for predicting the compressive strength of 3D-printed fiber-reinforced concrete using interpretable machine learning algorithms [29]. In conclusion, the proposed models could significantly reduce computational effort and the reliance on experimental trials in developing the mix design for 3D-printed concrete. A study explored the use of multi-target machine learning for designing sustainable steel fiber-reinforced concrete [30]. The framework achieved an error of less than 6.2% compared to advanced numerical simulations, underscoring its potential for optimal material identification in industrial implementations. In the literature, machine learning methods have been applied to civil engineering from various perspectives, consistently yielding reliable results.

The flexural behavior of steel bars and FRP-reinforced concrete beams was experimentally investigated with different composite materials [31]. The structural behavior of concrete beams reinforced with steel and GFRP bars was monitored using digital image correlation techniques [32]. An experimental study on the flexural behavior of corroded concrete beams reinforced with hybrid steel/GFRP bars was carried out [33,34].

Pre-cracked hybrid GFRP/steel RC beams were strengthened with CFRP sheets and tested, and strength estimation was performed [35]. The behavior of concrete beams reinforced with hybrid steel and FRP composites was investigated [36]. Three-dimensional nonlinear finite element analysis of concrete deep beam reinforced with GFRP bars was investigated [37]. The long-term deflection behavior of FRP-reinforced concrete beams was investigated [38].

Shear strengthening of polystyrene aggregate concrete beams with GFRP bars installed near the surface was explored [39]. The behavior and concrete strength of beams reinforced with varying ratios of glass fiber-reinforced polymer (GFRP) bars were analyzed [40]. An experimental investigation on the seismic performance of beam/column joints strengthened with GFRP reinforcements was conducted [41].

Nonlinear finite element analysis of high- and ultra-high-strength concrete beams reinforced with FRP bars was performed [42]. A three-dimensional (3D) nonlinear finite element analysis (FEA) model for shear critical glass fiber-reinforced polymer (GFRP) reinforced concrete beams was investigated [43].

A new practical equation for evaluating the shear capacity of FRP-RC slender beams by programming is proposed [44].

This study experimentally investigated the failure behavior and crack formation patterns of reinforced concrete beams with identical steel and GFRP bar reinforcement details at varying concrete compressive strengths.

Machine learning methods were used to estimate the deflection values of GFRP bar-reinforced concrete beams under ultimate load-carrying capacity, and the ML models that provided the best results were proposed for deflection estimation in GFRP bar-reinforced RC beams.

## 2. Materials and Methods

**Figure 1:**
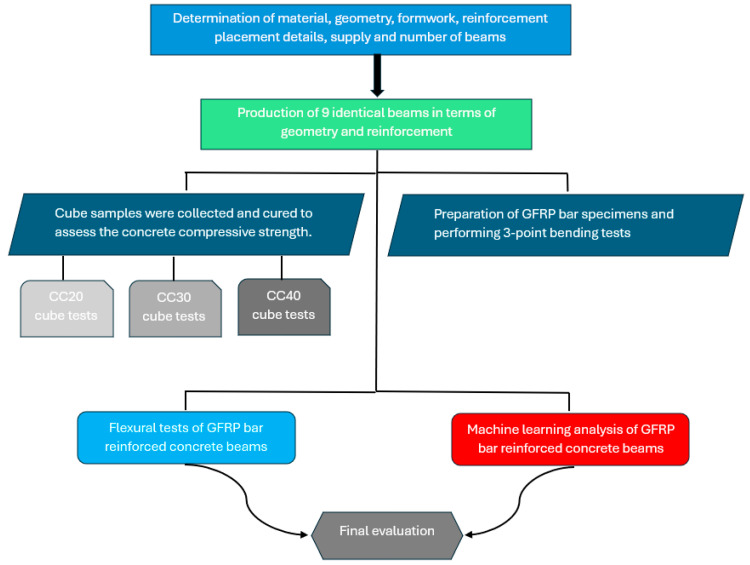
Program of experimental studies.

Reinforced concrete beams with GFRP bars in the tension zone are composed of three distinct materials: GFRP composite bars, steel bars, and concrete. The program of experimental studies is presented in Figure 1.

**Figure 2:**
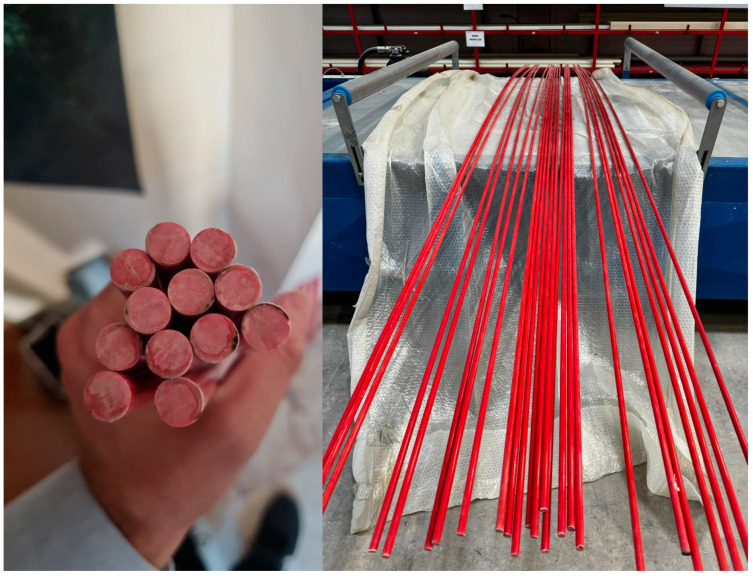
Cross-section and longitudinal GFRP bar reinforcement.

The GFRP bar reinforcements are composed of glass fiber, with 4800 Tex, and are bonded with polyester resin. Figure 2 presents the phi ϕ 10 mm cross-section and longitudinal GFRP bar reinforcement.

The mechanical properties of the GFRP bars used as longitudinal reinforcement obtained from the 3-point bending test are shown in Table 1. Three-point bending tests were conducted on GFRP bar test specimens until the ultimate bending load was reached, after which the load strength rapidly decreased. In the experiments, the GFRP bar lengths were set at 150 mm, while the distance between the supports was fixed at 100 mm.

**Figure 3:**
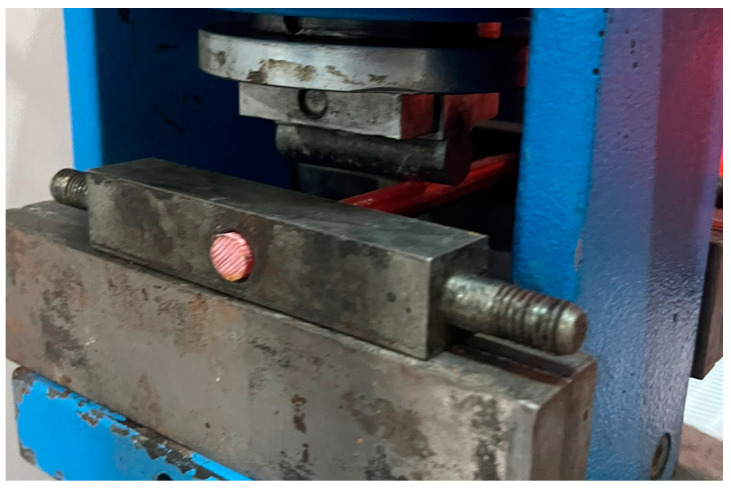
Three-point bending test setup of GFRP bar samples.

Figure 3 shows the 3-point bending test setup of GFRP bar samples.

Tests were performed with a loading rate of 30.5 Hz. The flexural behavior of the GFRP bar specimens under static loading was evaluated through an experimental investigation using a three-point bending test setup with the Yuksel Kaya Makina device.

**Figure 4:**
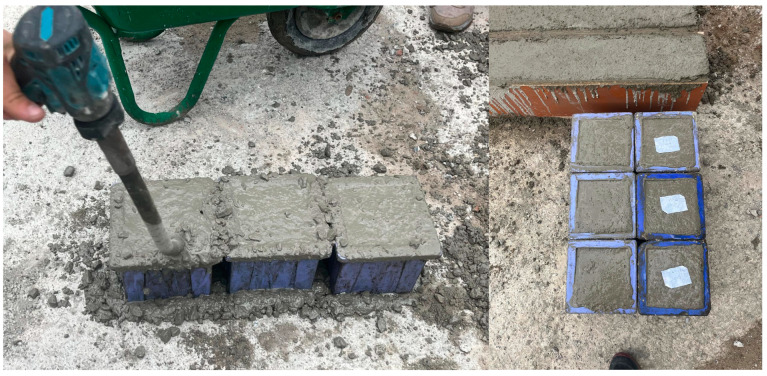
Vibration process and concrete cube samples taken from fresh concrete.

In this study, three groups of concrete classified by their compressive strength were utilized. Concrete cube samples taken from fresh concrete during the concreting of beams and the vibration process are presented in Figure 4.

The concrete compressive strength results for the low (C20), moderate (C30), and high (C40) cube concrete sample groups after 28 days of curing are provided in Table 2. Cube samples were placed in standard sample containers with dimensions of 150 × 150 × 150 mm.

The average compressive strength of three samples from each concrete strength class was used to determine the concrete compressive strengths for the C20, C30, and C40 concrete groups. In the study, two reinforcement steel bars with a diameter of φ10 mm and stirrups with a diameter of φ8 mm were used in the compression zone of the beams. Table 3 presents the mechanical properties of steel bar.

**Figure 5:**
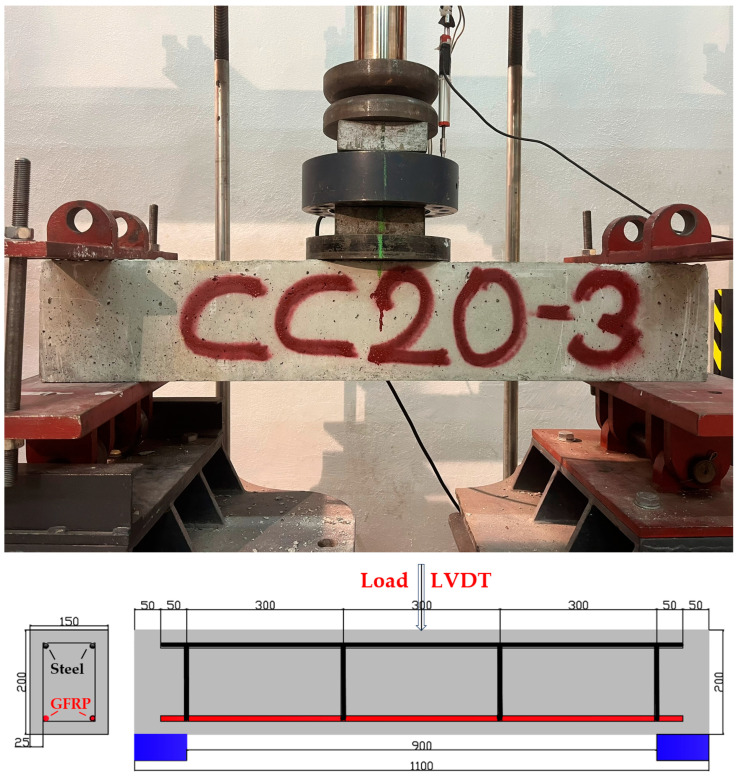
The 3-point bending test setup and the schematic view of GFRP-reinforced beams.

The 3-point bending test setup and the schematic view of GFRP-reinforced beams are presented in Figure 5. The nine GFRP bar-reinforced concrete beams fabricated are geometrically identical, featuring a width of 150 mm, a depth of 200 mm, and a length of 1100 mm. The longitudinal reinforcements in the compression zone of all reinforced concrete beams are steel bars with a 10 mm diameter, with two bars used per beam. The stirrups, also with a diameter of 8 mm, are spaced at 300 mm intervals along the beams. A concrete cover of 25 mm is provided on all sides around the wrapped area of each reinforced concrete beam. Three-point bending tests were conducted until the beams reached failure.

The three-point bending test method, along with bending moment/strength/stress relationships, deflection, and details for a rectangular cross-section beam, are presented below. Bending strength refers to the highest stress at the moment of rupture. Deflection (Δ) depends not only on the material but also on the cross-sectional configuration and unsupported length. The load-displacement response reveals a range of behaviors, including ductility, linear and nonlinear regions on the graph, and failure displacement. The formulas for maximum bending stress ($\sigma_{max}$), maximum bending moment ($M_{max}$), maximum shear stress for a solid section ($\tau_{max}$), and moment of inertia ($I_{solid}$) are provided in Equations (1)–(4). Key parameters, including bending moment, stress, deflection, and structural details for a rectangular cross-section beam, were analyzed. The bending strength was defined as the maximum stress experienced at the moment of rupture. Deflection (Δ) is dependent upon not only the material but also the configuration of cross-section and unsupported length. The load-displacement response indicates a diverse variety in many aspects, such as ductility, linear nonlinear area on the graph, and failure displacement. Max bending stress ($\sigma_{max}$), max bending moment ($M_{max}$), max shear stress for solid section ($\tau_{max}$), and moment of inertia ($I_{solid}$) formulas are presented in Equations (1)–(4).

(1) $$\sigma_{max}=\frac{Mc}{I}=\frac{3FL}{2wh^{2}}$$

(2) $$M_{max}=\frac{FL}{4}$$

(3) $$\tau_{max}=\frac{3F}{2wh}$$

(4) $$I_{solid}=\frac{bh^{3}}{12}$$

## 3. Experimental Investigation

**Figure 6:**
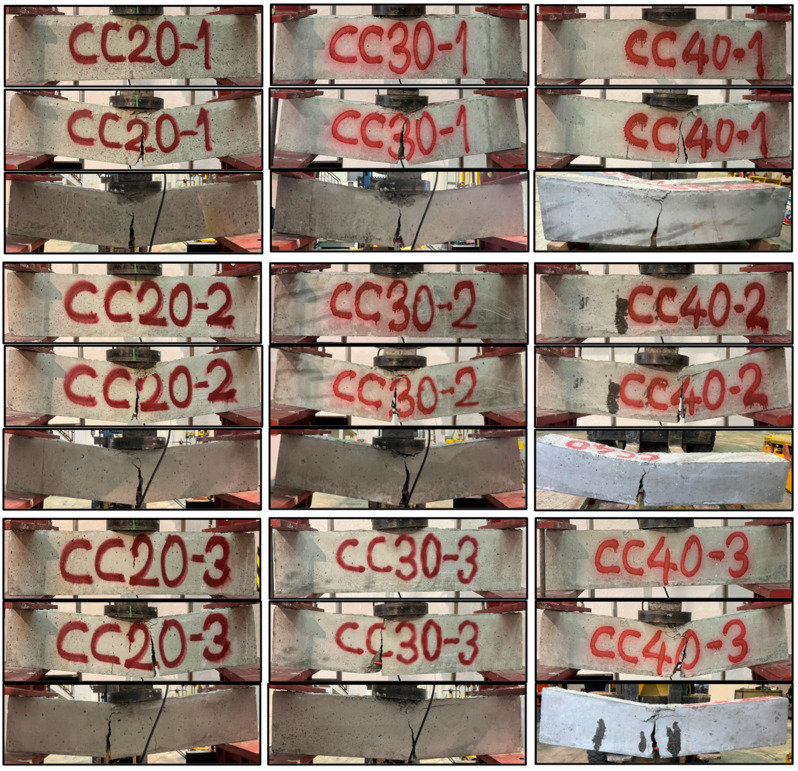
Three-point bending test of GFRP reinforced beams.

The experimental investigation of the reinforced concrete beams was conducted using a three-point bending test setup to evaluate their flexural behavior under static loading. The beams were simply supported with a span length of 900 mm, and a hydraulic testing machine applied controlled incremental loads at the midpoint of the span. A precision displacement transducer was installed at the midspan to record deflection, while a load cell accurately measured the applied forces. Each beam, with dimensions of 150 mm width, 200 mm depth, and 1100 mm length, was carefully positioned on the test apparatus, ensuring proper alignment with the supports. The load was distributed uniformly through a spreader beam to simulate realistic loading conditions. The test commenced with the gradual application of the load, and the corresponding deflections were recorded in real time. Crack formation and propagation were observed throughout the loading process, and failure modes were classified based on the crack patterns. The test was terminated once the beam reached its ultimate load-carrying capacity and exhibited visible failure. Critical data, including load-deflection responses, ultimate load, maximum deflection, and failure modes, were documented for each beam. Figure 6 indicates the front view of the nine beams before and after the three-point bending test, along with the post-test view of the rear section. The concrete compressive strengths of the nine test beams, which are produced identically in terms of reinforcement details and geometry, are different from each other as low (C20), moderate (C30), and high (C40). In order to clearly understand the flexural performance of each concrete compressive strength class, three identical GFRP bar-reinforced concrete beams were produced. This situation highlights the effect of concrete compressive strength on the bending performance of RC beams reinforced with GFRP bars. As a result of the tests, it was observed that all beams, regardless of the concrete compressive strength class, failed due to the crushing of the concrete in the compression zone of the beam and the formation of a large and thick bending crack starting from the middle bottom point of the beams.

**Figure 7:**
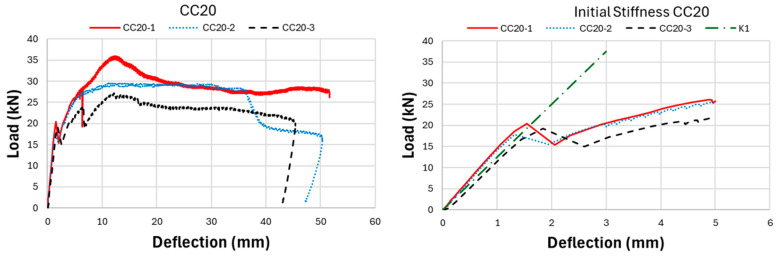
Experiment outcomes of CC20-GFRP bar-reinforced concrete beams: load deflection and initial stiffness.

**Figure 8:**
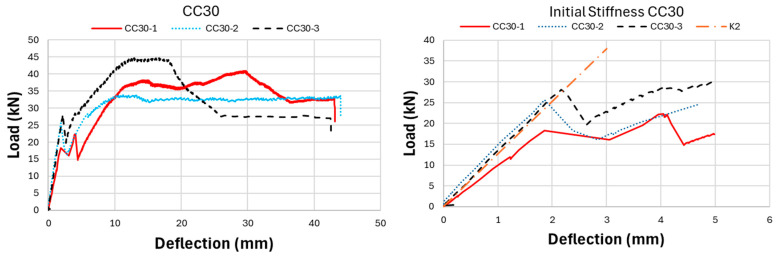
Experiment outcomes of CC30-GFRP bar-reinforced concrete beams: load deflection and initial stiffness.

**Figure 9:**
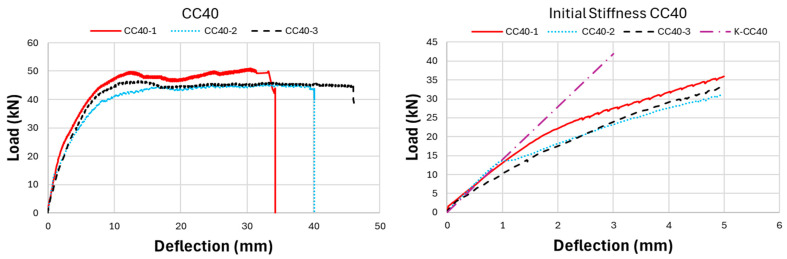
Experiment outcomes of CC40-GFRP-bar reinforced concrete beams: load deflection and initial stiffness.

**Figure 10:**
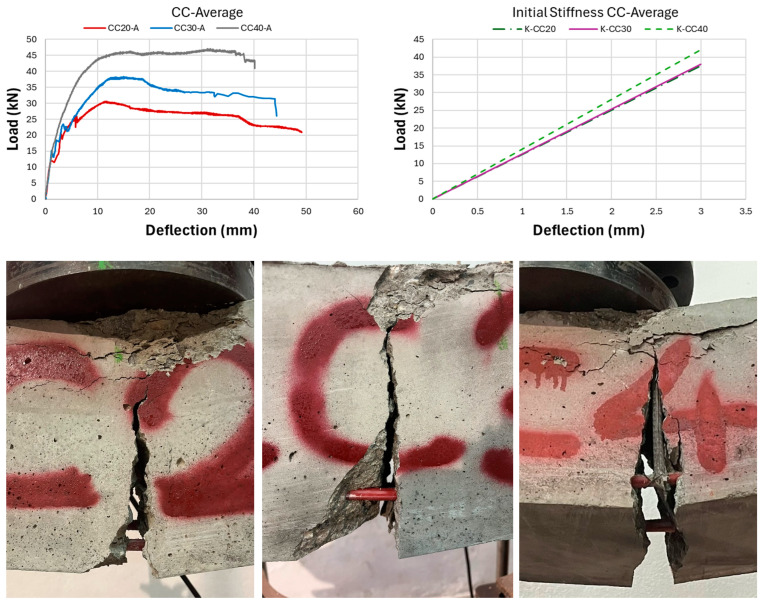
Experiment outcomes of CC-GFRP-bar reinforced concrete beams: average load deflection, initial stiffness, and beam fracture examples (CC20, CC30, and CC40 beams).

As a result of the experimental research, the ultimate load-carrying capacity of the CC20 beam group was obtained as approximately 29.13 kN on average, while this value was determined as 39.78 kN and 50.57 kN for CC30 and CC40, respectively. This reveals that CC40 beams have approximately 74% more load-carrying capacity compared to CC20 beams. Nonlinear load-deflection results of GFRP bar-reinforced concrete beams are presented in Figure 7, Figure 8, Figure 9 and Figure 10. When the three-point bending test results were examined, the CC40 beam group exhibited the highest ultimate load-carrying capacity, followed by the CC30 and the CC20 groups, as expected, with average values of approximately 50 kN, 40 kN, and 30 kN, respectively. In terms of deflection capacity, in contrast to the ultimate load-carrying capacity, the most ductile beams were the CC20 group, followed by CC30 and CC40 with approximately the following values: 49, 44, and 40 mm, respectively.

Figure 7 presents the load-deflection curves of the CC20 group and the initial stiffnesses belonging to these curves. As presented in Figure 7, the CC20-1 beam reached the highest ultimate load-carrying capacity in the CC20 group with a value of 36 kN. Following CC20-1, the CC20-2 beam exhibited the second-highest load-carrying capacity with 29.7 kN, while the CC20-3 beam exhibited the lowest value with 27.1 kN. The deflection values of the CC20 group beams are 51.7 mm, 50.3 mm, and 45.4 mm for CC20-1, CC20-2, and CC20-3, respectively. Figure 8 presents the three-point bending test results of the CC30 group. In the CC30 group, the CC30-3 beam indicates the highest load-carrying capacity with 44.64 kN, followed by the CC30-1 and CC30-2 beams with 40.9 and 33.8 kN values, respectively. In terms of deflection values, the CC30-2 beam reaches the largest deflection value with 44 mm, followed by CC30-1 and CC30-2 with values of 43.1 mm and 42.5 mm, respectively. Figure 9 presents the experimental results of the CC40 group, which has the highest ultimate load-carrying capacity and the lowest deflection value. When the CC40 group was examined in terms of ultimate load-carrying capacity, the CC40-1, CC40-3, and CC40-2 beams exhibited the highest to lowest values of 60 kN, 46.2 kN, and 45.5 kN, respectively. When the deflection behavior of the CC40 group was evaluated, the CC40-3 beam displayed the highest ductility, showing a deflection of 46.1 mm. This was followed by the CC40-2 and CC40-1 beams, with deflections of 40.1 mm and 34.3 mm, respectively. Figure 10 illustrates the average values for the beams from the three groups, providing a clearer comparison of the flexural behavior of GFRP-reinforced concrete beams with low (CC20), moderate (CC30), and high (CC40) concrete compressive strengths. This visual aid enhances understanding of how varying concrete strengths influence the flexural performance of the beams. As shown in Figure 10, the load-carrying capacity of GFRP bar-reinforced concrete beams increases with higher concrete compressive strength. However, in contrast to the ultimate load capacity, both the deflection values and ductility decrease as the concrete compressive strength rises. The CC40 group exhibited a higher load-carrying capacity but a more brittle failure behavior, whereas the CC30 and CC20 groups achieved a lower load-carrying capacity but exhibited a more ductile behavior. The initial stiffness values of GFRP bar-reinforced concrete beams, as shown in the graphs in Figure 7, Figure 8, Figure 9 and Figure 10, increase with the rise in concrete compressive strength. Additionally, the initial stiffness of the CC30 group displays a slightly steeper slope compared to the CC20 group, with this trend becoming more pronounced in the CC40 beam group. In this case, it is interpreted that the difference in concrete compressive strength between CC40 and CC30 beams is greater than the difference in concrete compressive strength between CC30 and CC20. Exactly, this can be interpreted as the increasing concrete compressive strength having a direct positive effect on the initial stiffness of the beams. As concrete strength rises, it enhances the beam’s ability to resist deformation under load, which is reflected in the steeper slope of the load-deflection curve, especially in the CC40 group.

Table 4 presents the results of flexural tests on nine GFRP bar-reinforced concrete beams. The failure modes of the beams are categorized by color, ranging from green (ductile behavior) to red (brittle behavior). The beams are color-coded green, indicating that they displayed flexural cracking and ductile behavior without exhibiting shear cracks that could lead to sudden failure. The average experimental data for each concrete compressive strength group, as shown in Table 4, are listed in the rows labeled with an “A” code. Notably, the beams in the C40 series, which have a high concrete compressive strength, demonstrate the lowest initial crack load among the different GFRP-reinforced beam groups.

## 4. Machine Learning Analysis

**Figure 11:**
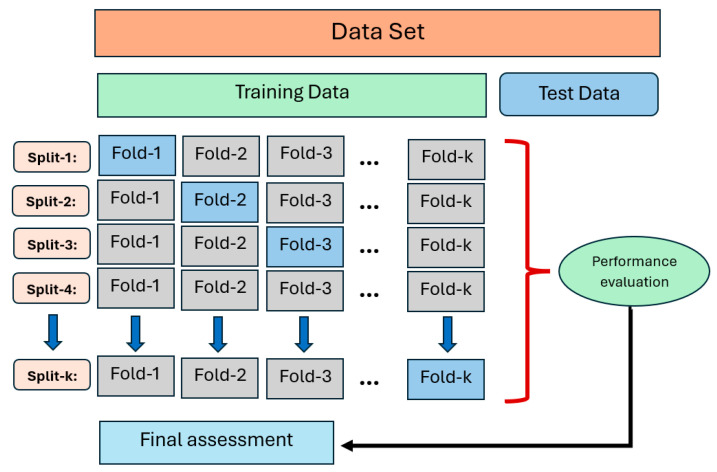
K-fold cross-validation method (K = 10).

The machine learning (ML) methodology implemented in this study provides a detailed and accurate prediction framework for the deflection behavior of GFRP bar-reinforced concrete (RC) beams under varying load conditions. A total of eighteen regression models were employed to predict deflection values based on load-deflection experimental datasets for beams reinforced with GFRP (glass fiber-reinforced polymer) bar across three concrete compressive strength levels: low (C20), moderate (C30), and high (C40). The ML analysis was performed using the PyCaret library, employing an 80:20 training-to-testing data split and 10-fold cross-validation to ensure the robustness and generalization of the models. Figure 11 presents the K-fold cross-validation method (K = 10).

**Figure 12:**
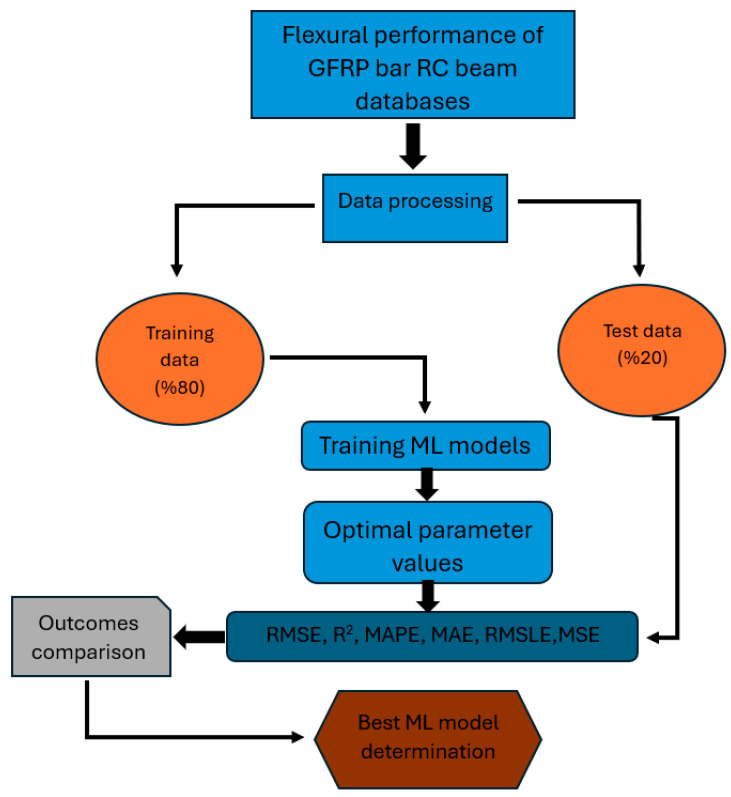
Overall structure of the developed ML models.

The dataset, generated from the load-deflection responses of 9 GFRP bar RC beams tested experimentally, was used to train and validate various ML regression models. Python’s PyCaret library was utilized for automating the ML workflow. The dataset was split into 80% training data and 20% testing data, with key input parameters including concrete compressive strength (*fc′*), beam dimensions, reinforcement ratios, and load values, while the target variable was the midspan deflection (Δ). A total of 18 regression models were evaluated, including Gradient Boosting Regressor, Light Gradient Boosting Machine, K Neighbors Regressor, Ada Boost Regressor, and Extra Trees Regressor, among others. The models were assessed using 10-fold cross-validation and performance metrics such as Root Mean Squared Error (RMSE), Mean Absolute Error (MAE), and the coefficient of determination (R^2^). Hyperparameter tuning was conducted to optimize the models for higher accuracy and lower prediction errors. By integrating experimental data with ML analysis, the study provided a robust framework for predicting load-deflection behavior, validating experimental findings, and offering insights for optimizing structural design parameters. Figure 12 represents the model of the ML structure used in the present study.

The 18 machine learning regression models utilized in the ultimate load-deflection curve capacity research obtained from the experimental results of steel and GFRP bar-reinforced concrete beams are displayed in Table 5. Various concrete compressive strengths, reinforcement material types, beam cross-sectional width, height, effective depth, and reinforcement ratios are considered in the research. In statistical computation techniques, to interpret the effectiveness of a machine learning algorithm, frequently utilized metrics include Root Mean Squared Log Error (RMSLE), Root Mean Square Error (RMSE), Mean Absolute Error (MAE), Mean Squared Error (MSE), Coefficient of Determination (R^2^), and Mean Absolute Percentage Error (MAPE). These assessment metrics objectively evaluate the degree of proximity between the ML regression model’s forecasts and the actual values.

In a competently performing model, inferior values of RMSE, MAE, and MAPE exhibit better accomplishment, while R^2^ values closer to 1.00 display better proper [26]. The evaluation equations are displayed as Equations (5)–(8) [26]:

(5) $$RMSE=\sqrt{\frac{\sum_{i=1}^{n}{\left(x_{i}-{x^{\prime }}_{i}\right)}^{2}}{N}}$$

(6) $$MAE=\frac{1}{N}\sum_{i=1}^{n}\left|x_{i}-{x^{\prime }}_{i}\right|\;$$

(7) $$R^{2}=1-\sum_{i=1}^{n}\;\frac{{\left(x_{i}-{x^{\prime }}_{i}\right)}^{2}}{\sum_{i=1}^{n}\;(x_{i}-{x^{\prime }}_{i})}$$

(8) $$MAPE=\sum_{i=1}^{n}\frac{1}{N}\left|{x^{\prime }}_{i}-\frac{x_{i}}{{x^{\prime }}_{i}}\right|$$

Estimation of strength, ultimate load carrying capacity, and deflection values of composites using Pycaret regression analysis have been studied to a limited extent, but the analysis outcomes are highly consistent [45]. MAE, MSE, R^2^, RMSE, and RMSLE values were calculated to estimate the ultimate load-deflection curves of GFRP bar-reinforced concrete beams using 18 different regression analyses. The regression models, with average results from 10-fold cross-validation, were considered for evaluating recognition performance. Table 6 presents the input and output values of important parameters utilized in machine learning regression models. From the input values presented in Table 6, concrete compressive strengths (*fc′*) are taken into consideration separately for the C20, C30, and C40 series for the 28-day cube sample results of concrete. Tensile yield strength (fy) values for steel reinforcement are kept constant at 420 MPa. The width, height, length, and effective depth values of the beams are also considered constant. GFRP bar tensile strengths (ff) are considered between 469 MPa and 635 MPa. The ultimate high load (F) carrying capacities of reinforced concrete beams obtained in 3-point bending tests are the lowest, 21.70 kN, and the highest, 60.01 kN, in ML regression analyses. Reinforcement ratios (ρf, ρb) are also included in the analysis from lowest to highest value.

**Figure 13:**
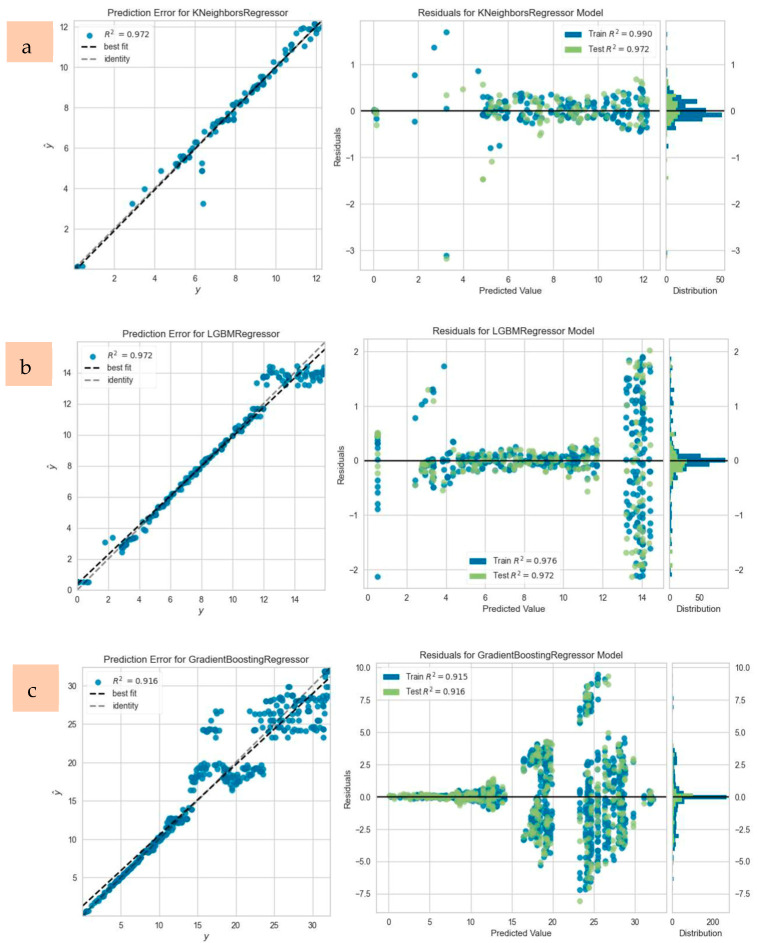
Prediction error and residual plots of (**a**) CC20-1, (**b**) CC30-3, (**c**) CC40-2.

The regression models were evaluated based on their ability to predict deflection values with high accuracy, measured primarily through the R^2^ metric. For each beam, the model yielding the most accurate deflection predictions under ultimate load conditions was identified. Additionally, prediction errors and residual plots were generated to assess the alignment between experimentally measured and predicted values. Representative prediction error and residual plots for selected beams, such as CC20-1, CC30-3, and CC40-2, are displayed in Figure 13. The PyCaret library facilitated accuracy evaluation through six statistical metrics, including *R*^2^, RMSE, MAE, and MAPE, providing a comprehensive understanding of the models’ predictive capabilities [45].

The cross-validation results, presented in Table 7, identified the Gradient Boosting Regressor (GBR) as the most accurate model for predicting deflection under ultimate load conditions. For instance, the CC30-2 beam achieved the highest *R*^2^ value of 0.9850, highlighting the exceptional performance of GBR in capturing the behavior of GFRP bar-reinforced beams with moderate concrete compressive strength. Conversely, the lowest *R*^2^ value was recorded for the CC40-1 beam (GFRP-reinforced, high-strength concrete), reflecting greater variability in its load-deflection response. Overall, the average *R*^2^ value for all beams was 0.915, indicating excellent agreement between the experimental data and ML predictions.

**Figure 14:**
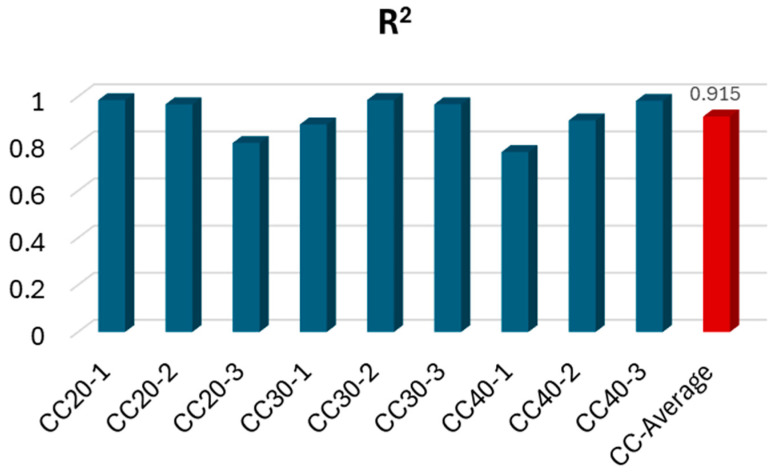
The results of the machine learning regression analysis performed for the deflection.

The results of the ML regression analysis are summarized in Figure 14, which illustrates the high predictive accuracy of the models for deflection capacities across all beam configurations. Among the 18 models tested, the GBR and ADA consistently delivered the best results, followed by the Light Gradient Boosting Machine and K Neighbors Regressor. These models demonstrated their ability to handle the nonlinear and material-dependent behavior of GFRP bar RC beams effectively. The K Neighbors Regressor, while less frequently the top-performing model, provided the second highest R^2^ value for certain cases, such as CC20-1, showcasing its suitability for datasets with minimal variability. While the average of the machine learning analysis results of all composite bar reinforced concrete beams is shown in the red column, the analysis results for each beam are presented in blue.

**Figure 15:**
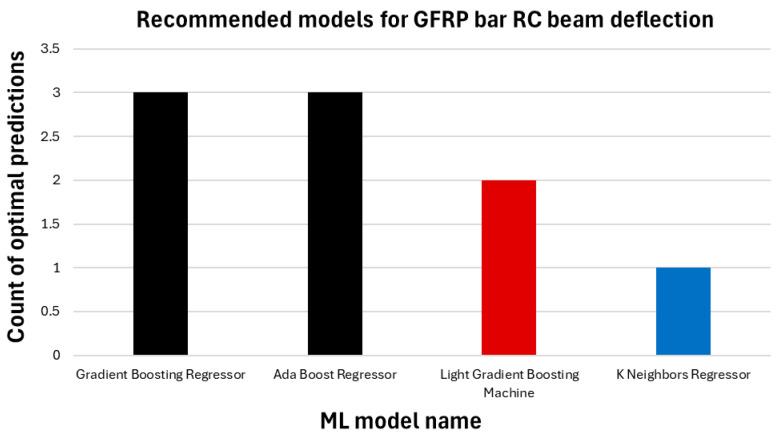
Prediction numbers of the most optimal predictive ML regression models.

Figure 15 provides a comparative overview of the prediction capabilities of the various ML models. It underscores the dominant performance of the GBR and ADA model, which accurately predicted deflection values for the largest number of beams. Other models like the Light Gradient Boosting Machine and K Neighbors Regressor followed closely, each excelling in specific scenarios. Figure 15 illustrates the ML regression models, each represented in a different color based on the number of predictions correctly. The black model achieved the highest number of correct predictions, with three successful predictions. The red model made the best prediction twice, while the blue model had the fewest correct predictions, with only one.

## 5. Conclusions

This study combined experimental testing and machine learning (ML) techniques to analyze the flexural performance of GFRP (glass fiber-reinforced polymer) bars across low (C20), moderate (C30), and high (C40) concrete compressive strengths. The following summarizes the key insights and recommendations from the study:

1. GFRP bar-reinforced concrete (RC) beams demonstrated ductile behavior with gradual energy dissipation and flexural cracking.

2. No shear cracks occurred in beams produced with GFRP bar reinforcement, even at low (C20) concrete compressive strengths.

3. The ultimate load-bearing capacity of CC40 RC beams was approximately 74% higher compared to CC20 RC beams.

4. Regardless of the concrete compressive strength class, GFRP bar-reinforced concrete beams do not experience shear cracks or sudden failure, which are significant advantages of using GFRP bar reinforcement.

5. The GFRP bar surface can be improved to increase concrete and GFRP bar adhesion.

6. The study found that all beams, regardless of concrete compressive strength (low, moderate, or high), failed due to crushing in the upper part of the beam and bending cracks at mid-span. The GFRP bar reinforcement in the tension regions did not yield. It is recommended to further test the failure behavior of beams with higher concrete compressive strength using the same experimental method and beam reinforcement details.

7. The test results indicated that as the concrete compressive strength class increases, the ultimate load-carrying capacity of the beams also increases. However, the deflection value of the midpoint of the beams and the ductility of the beams decreased as the concrete compressive strength increased.

8. The research results obtained through machine learning analysis on GFRP bar-reinforced concrete beams exhibited a high degree of consistency with machine learning findings for deflection in steel rebar-reinforced concrete beams from the literature [26]. The deflection results for GFRP bar-reinforced RC beams showed an accuracy of 91.5%, while the corresponding value in the literature for steel-reinforced RC beams was 95%.

9. This study demonstrates that machine learning analysis can be effectively applied to load-deflection calculations of GFRP bar-reinforced concrete beams.

10. Gradient Boosting Regressor and Ada Boost Regressor machine learning models are proposed for the prediction of the flexural capacity of GFRP bar-reinforced concrete beams.

11. It is suggested that machine learning regression models be more extensively applied in structural engineering, particularly for RC beam calculations.

12. GFRP bar-reinforced cases of ultra-high strength reinforced concrete beams are recommended for future studies.

## Figures and Tables

**Table 1:** Mechanical properties of GFRP bars.

GFRP Bar Diameter (mm)	Cross-Sectional Area (mm^2^)	GFRP Bar Sample Length (mm)	Max Bending Load (kN)	Max. Bending Strength (MPa)	Ultimate Deflection Δ (mm)	Weight (g/cm)	Support Distance (mm)
10	78.54	150	3.2	40.11	4.67	1.59	100

**Table 2:** Mechanical properties of concrete groups.

Concrete Group	fc (Cylinder-MPa)	fctk (MPa)
C20	28.1	1.85
C30	35.9	2.10
C40	44.8	2.30

**Table 3:** Mechanical properties of steel bar.

Material	Modulus of Elasticity (E)	Density ρ (kg/m^3^)	Poisson Ratio ν
Steel rebar	200 GPa	7850	0.3

**Table 4:** The results detail of flexural tests.

Series	Beam No.	GFRP Bar RC Beam Crack Form and Failure Behavior	Initial Cracking Load, Fcr (kN)	Failure Load, Fexp (kN)	Maximum Mid Span Deflection, Δ exp (mm)	Failure Moment, Mexp (kN.m)
CC20	CC20-1	FC-ductile	20.47	36.01	51.7	16.20
	CC20-2	FC-ductile	17.76	29.71	50.3	13.40
	CC20-3	FC-ductile	19.23	21.70	45.4	9.77
	CC20-A	-	19.15	29.13	49.1	13.12
CC30	CC30-1	FC-ductile	18.35	40.91	43.1	18.41
	CC30-2	FC-ductile	25.98	33.80	42.5	15.21
	CC30-3	FC-ductile	27.23	44.64	44	20.09
	CC30-A	-	22.72	39.78	43.2	17.90
CC40	CC40-1	FC-ductile	43.37	60.01	34.3	27
	CC40-2	FC-ductile	41.93	45.50	40.1	20.48
	CC40-3	FC-ductile	39.22	46.20	46.1	20.79
	CC40-A	-	41.51	50.57	40.2	22.76

FC: Flexural Crack. Ductility behavior decreases from green to red.

**Table 5:** ML regression models.

ML Model No.	ML Regression Model Name	ML Model Name Code
1	Gradient Boosting Regressor	gbr
2	K Neighbors Regressor	knn
3	Ada Boost Regressor	ada
4	Random Forest Regressor	rf
5	Light Gradient Boosting Machine	lightgbm
6	Extra Trees Regressor	et
7	Decision Tree Regressor	dt
8	Lasso List Angle Regressor	llar
9	Ridge Regression	ridge
10	Bayesian Ridge	br
11	Orthogonal Matching Pursuit	omp
12	Elastic Net	en
13	Least Angle Regression	lar
14	Lasso Regression	lasso
15	Linear Regression	lr
16	Huber Regression	huber
17	Passive Aggressive Regressor	par
18	Dummy Regressor	dummy

**Table 6:** Statistical details of the parameters in the database.

Feature	Type	Cmin	Cmax	Ave
fc′ (MPa)	Input	33.91	53.38	41.86
fy (MPa)	Input	420	420	420
ff (MPa)	Input	469	635	562
b (mm)	Input	150	150	150
h (mm)	Input	200	200	200
d (mm)	Input	162	162	162
L (mm)	Input	1100	1100	1100
ρf (%)	Input	0.6	0.99	0.79
ρb (%)	Input	2.17	3.57	2.85
F (kN)	Input	21.70	60.01	39.83
Δ (mm)	Output	34.3	51.7	44.2

**Table 7:** Performance metrics of ML model.

Beam Name	Best Predictive ML Model	MAE	MSE	RMSE	R^2^	RMSLE	MAPE
CC20-1	K Neighbors Regressor	0.2351	0.1502	0.3402	0.9847	0.0632	0.1041
CC20-2	Gradient Boosting Regressor	0.3584	0.2646	0.5112	0.9667	0.0583	0.0837
CC20-3	Ada Boost Regressor	0.9490	1.3719	1.1666	0.8026	0.1687	0.3246
CC30-1	Ada Boost Regressor	1.9744	7.7575	2.7716	0.8814	0.1750	1.1009
CC30-2	Gradient Boosting Regressor	0.2340	0.1993	0.4366	0.9850	0.0534	0.0926
CC30-3	Light Gradient Boosting Machine	0.4881	0.5921	0.7634	0.9671	0.1267	0.4986
CC40-1	Ada Boost Regressor	3.0847	17.7252	4.2083	0.7641	0.2540	0.4381
CC40-2	Gradient Boosting Regressor	1.8281	7.7855	2.7791	0.8985	0.1243	0.1692
CC40-3	Light Gradient Boosting Machine	0.2745	0.2005	0.4335	0.9817	0.1012	0.5880

## Data Availability

The experimental data is available upon request due to privacy reasons.
